# Correction: Lactate and H3K18 Lactylation Contribute To the Exacerbation of Acute Pancreatitis by Modulating NCOA4-mediated Ferroptosis

**DOI:** 10.1007/s10753-026-02477-8

**Published:** 2026-03-14

**Authors:** Zi-Yu Han, Xue-Ying Ma, Shi-Ya Ma, Zhao-Jian Shen, Zhong-Hua Lu, Yun Sun, Wei-Li Yu

**Affiliations:** The First Department of Critical Care Medicine, The Second Affiliated Hospital of Anhui Medical University, Hefei, 230601 China

**Correction: Inflammation (2026) 49:18**

**https://doi.org/10.1007/s10753-025-02361-x**

The originally published version of this article contained mistakes, and the author would like to correct it.

The online date of the published article was wrongly written as November 25, 2026. The correct online date should be November 25, 2025.

The original article has been corrected.

